# Investigation of Microstructure and Mechanical Properties of High-Depth-to-Width-Ratio Horizontal NG-GMAW Joint for S500Q Steel

**DOI:** 10.3390/ma17092056

**Published:** 2024-04-27

**Authors:** Ruiyan Jia, Haichao Li, Fangkai Wei, Yufei Zhou, Weizan Duan, Kuiliang Zhang, Zhenglong Lei

**Affiliations:** 1 State Key Laboratory of Advanced Welding and Joining, Harbin Institute of Technology, Harbin 150001, China; jry@hec-china.com (R.J.); leizhenglong@hit.edu.cn (Z.L.); 2 Harbin Electric Machinery Company Limited, Harbin 150001, China; weifangkai@hec-china.com (F.W.); zyf@hec-china.com (Y.Z.); wzduan2010@163.com (W.D.); zhangkl@hec-china.com (K.Z.)

**Keywords:** NG-GMAW, S500Q steel, tensile strength, impact toughness, microstructure

## Abstract

A novel high depth-to-width ratio of 15:1 narrow-gap gas metal arc welding technique was developed for the welding of S500Q steel in a horizontal butt joint. The bead arrangement of the I groove was optimized to produce a high-quality connection with the upper sidewall of the joint. The microstructure and mechanical properties were observed and evaluated by optical microscopy, scanning electron microscopy, tensile testing, and micro-hardness and impact toughness testing at 1/5, 2/5, 3/5, and 4/5 thickness of the joint. The 3/5 T position exhibited the highest strength, which was attributed to the presence of finer carbide precipitates. The highest micro-hardness appeared at 4/5 T. The highest impact toughness appeared at 3/5 T. The formation of coarse granular bainite was the major reason for the decrease in impact toughness in other regions. A microscopic fracture at 1/5 T and 3/5 T was further analyzed. It was observed that the width of the fibrous zone at 3/5 T was significantly larger than that at 1/5 T. The radial zones at 1/5 T were observed to exhibit cleavage, with secondary cracks on the fracture surface.

## 1. Introduction

A pumped storage power station can freely choose the direction of energy conversion. It can flexibly select operating conditions, referring to the demand of the power grid, and has functions of peak shaving, valley shaving, frequency regulation, emergency reserve, and optimal power supply, which have been vigorously developed and widely used.

The stay ring is an important part of a pumped storage unit [1]. The stay ring requires high-strength and ultra-thick welded structural steel materials. S500Q is a 500 MPa grade low-alloy, high-strength steel developed in China, which is widely used in hydropower equipment [2]. As the units increase in capacity, the stay ring experiences an increase in thickness. Narrow-gap gas metal arc welding (NG-GMAW) is an advanced, highly efficient technology for joining thick-section metals. NG-GMAW has the advantages of energy saving, material saving, high efficiency, and a low welding heat input, stress deformation, thermal damage, etc. [3,4]. Common defects, such as a lack of fusion at groove sidewalls and porosity in weld joints, are still hard to avoid, especially for joining thick-section metals. Researchers studied the formation characteristics of welds and the influence of the oscillation width and sidewall dwell time on the formation process of narrow-gap welding [5,6,7]. The basic principle of the oscillating arc is to use the oscillation of the entire welding torch, the bending of the welding wire, the bending of the contact, the alternating magnetic fields, and other methods to make the arc oscillate back and forth on both sides of the groove to ensure sidewall fusion [8,9,10,11]. Liu and Fang designed a narrow-gap gas-shielded three-wired indirect arc welding torch, which greatly improved welding efficiency [12,13,14]. After the narrow-gap three-wired indirect arc, a tungsten electrode arc was used to make the convex deformation of the weld surface concave. Researchers studied the technology of planar position laser arc composite welding [15,16,17,18]. For narrow-gap welding, the optimization of welding process parameters, the oscillating arc, rotating arc, multi-wire welding, composite welding, and other specific methods have been studied to greatly optimize the weld formation and improve welding efficiency [19,20,21].

**Figure 1:**
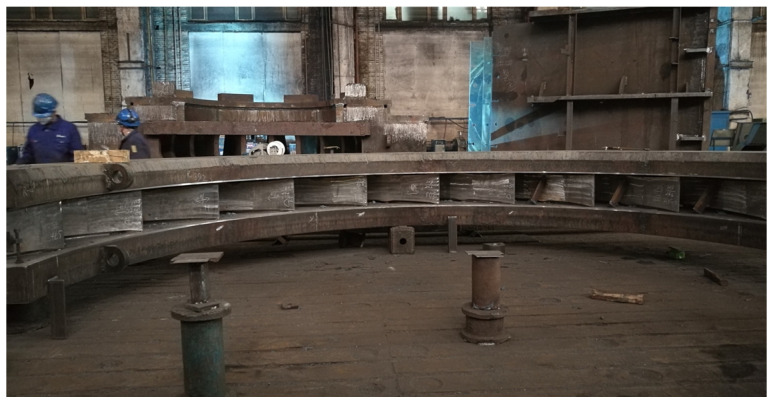
The stay ring in the horizontal welding position.

In stay ring welding, as depicted in Figure 1, which is difficult to turn over, it is necessary to adopt a horizontal welding position. In the horizontal-position welding process, the key problem is weld defects induced by the downward motion of the molten metal and poor weld formation [22,23]. Guo showed that the rotating arc process could be beneficial for the molten pool control of narrow-gap horizontal welding [24]. Cui controlled the horizontal weld formation of a molten pool by reducing the heat input and changing the effect of arc force and droplet impact on the molten pool [25]. However, the efficiency is greatly reduced. The rotating arc welding process must be carried out under the precise control of a small current (200–220 A) and the position of the welding torch. Otherwise, a slight deviation may lead to the instability of the welding process and even cause a huge disaster [26,27]. The multi-layer double-pass welding process has low position-accuracy requirements, good adaptability to groove size tolerance and thermal shrinkage during the welding process, a relatively wide selection range of welding parameters, a reduced production cost and operational complexity, and considerable advantages in practical applications.

However, the existing research studies are focused on the technical realization of thick-plate connections and the microstructure of specific locations, including the base metal (BM), weld metal (WM), and heat-affected zone (HAZ). There are few studies on narrow-gap welding for a high-depth-to-width-ratio horizontal NG-GMAW of more than 200 mm thickness. No attention has been paid to the microstructural inhomogeneity of the weld joint along the thickness direction and its influence on the properties of the weld joint. This paper aims to study the formation of inhomogeneous microstructures and analyze the effect of mechanical properties on the welding performance of S500Q steel with a 225 mm thickness. It is expected that the findings will contribute to promoting a better understanding of the microstructure development and mechanical properties of the NG-GMAW ultra-thick plates of the stay ring.

## 2. Materials and Methods

**Figure 2:**
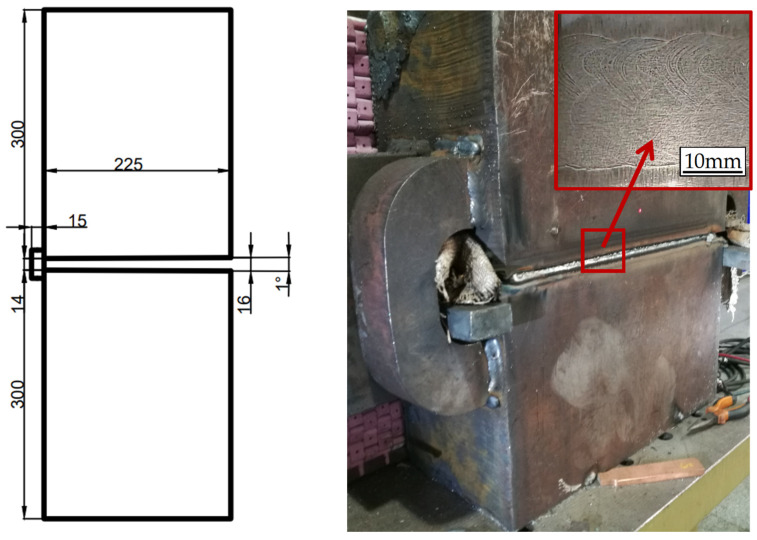
Groove size of high-depth-to-width-ratio horizontal NG-GMAW (mm).

The dimensions of the welded specimen were 500 mm × 300 mm × 225 mm. The narrow-gap welding assembly dimensions were 500 mm × 614 mm × 225 mm, with an approximate bevel surface angle of 1°, as shown in Figure 2. The groove of the welded specimen was type I, with a 14–16 mm gap and a depth-to-width ratio of 15:1. The BM was S500Q steel, which exhibited a high strength and toughness [28]. S500Q is a European standard material grade, which specifies the mechanical properties and chemical composition of steel plates below 150 mm thickness in EN10025-6:2004 [29]. In order to match the requirement for thick plates above 200 mm of the stay ring, this paper applied the S500Q steel developed in China. The S500Q steel was manufactured by Nan Yang Han Ye Special Steel Co., Ltd., Nanyang, China. Table 1 presents its mechanical properties, and Table 2 displays its chemical composition [30]. According to EN10025-6:2004, S500Q steel evaluates the impact toughness at −20 °C without special requirements. Due to the power station being located in the northeast of China and the ambient temperature being lower than −20 °C, the impact toughness at −40 °C was studied in this paper.

**Figure 3:**
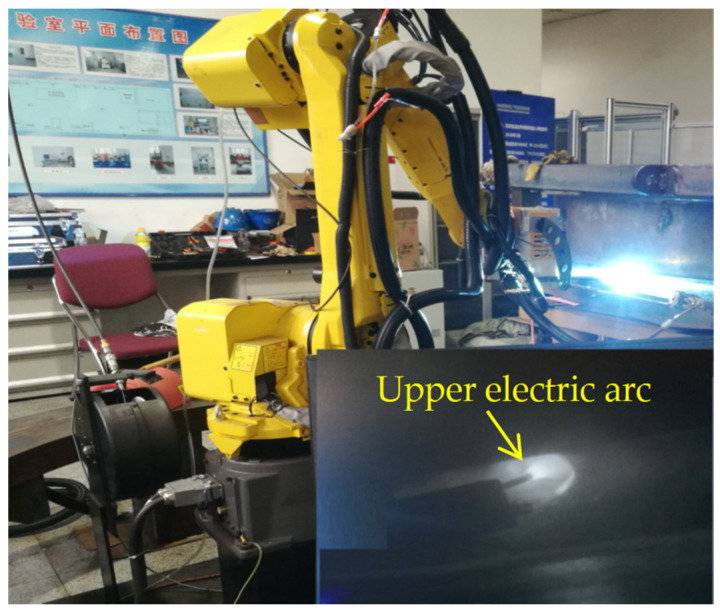
The welding system used in a horizontal joint.

**Figure 4:**
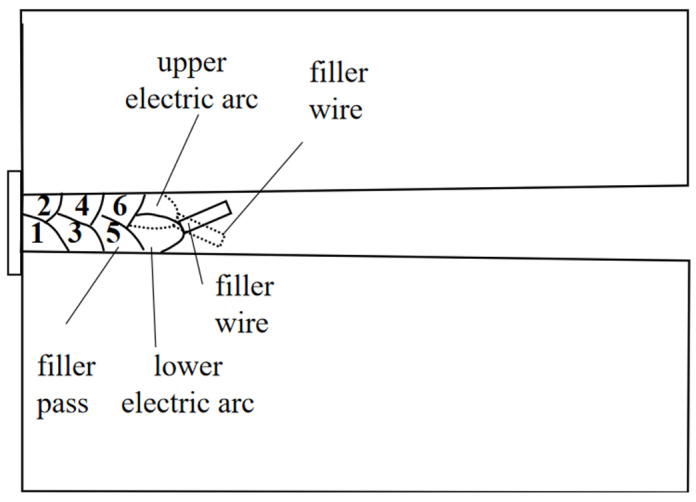
The bead arrangement and electric arc position characteristics.

AWS ER90S-G wire with a diameter of 1.2 mm was used as the welding wire. Table 3 and Table 4 present its chemical composition and mechanical properties, respectively. As shown in Figure 3, the welding system was composed of an NG-GMAW torch and a Fronius (TransPuls Synergic-500i, Fronius, Pettenbach, Austria) welding power supply equipped with a Fanuc M-20iA robot (Fanuc, Yamanashi, Japan). The bead arrangement is illustrated in Figure 4. The arrangement of two welding paths per layer was adopted, and the upper weld path was supported by the lower weld path.

**Figure 5:**
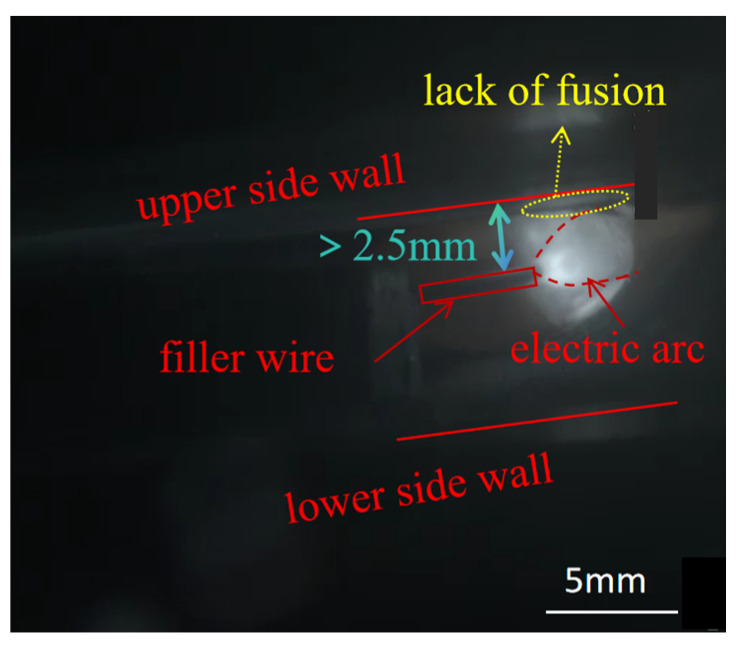
Appearance of a melt pool lack of fusion.

**Figure 6:**
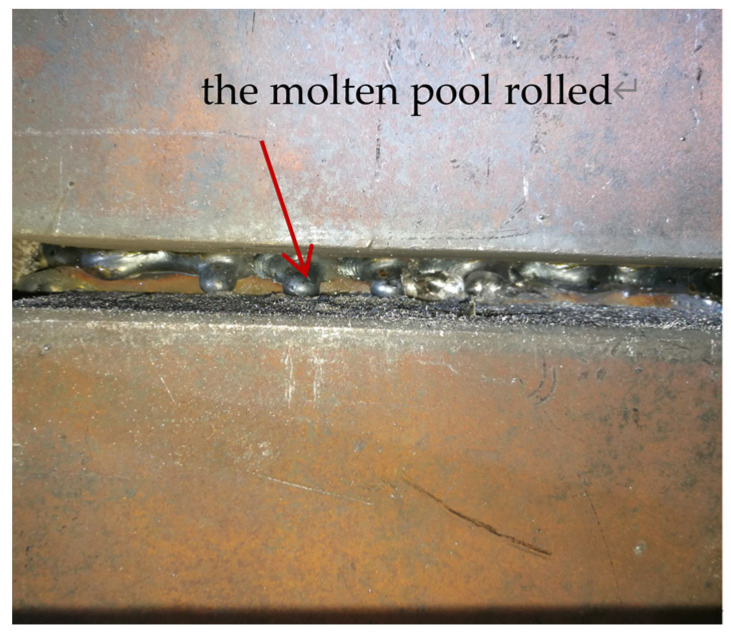
The molten pool rolled.

In narrow-gap horizontal welding, the filler wires of the upper weld path and lower weld path were fixed at an angle and did not swing. When the distance between the wire and the sidewall was greater than 2.5 mm, it was easy to produce a lack of fusion, as shown in Figure 5. When the distance between the wire and the upper sidewall was smaller than 1.5 mm, it caused the molten pool to roll and flow, as shown in Figure 6.

Thus, the distance from 1.5 to 2.5 mm was used in this study to promote a high quality of high-depth-to-width-ratio horizontal NG-GMAW for S500Q steel, and the detailed parameters were as shown in Table 5.

**Figure 7:**
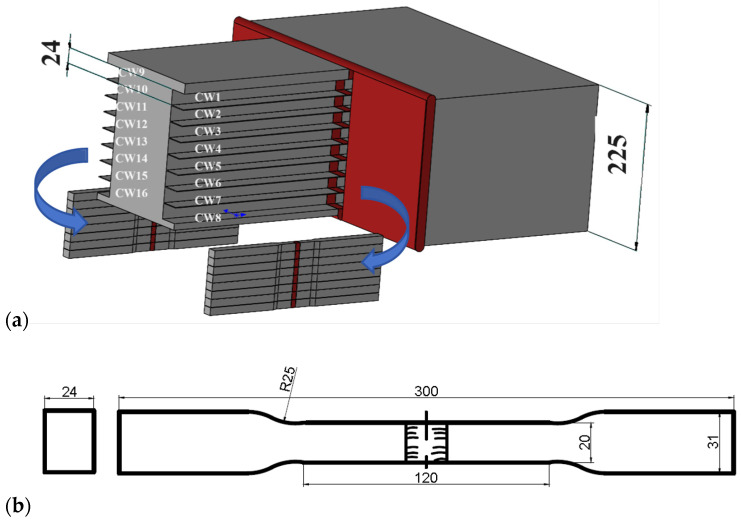
The tensile specimens: (**a**) positions; (**b**) dimensions (unit: mm).

According to ISO 4136-2022 [31], the tensile specimens were prepared at room temperature (20 °C). The layout of the tensile specimens is shown in Figure 7a. In the WM, there were 16 through-thickness cross-weld tensile specimens marked as CW1–16. Specimens CW1 and CW9 were located 15 mm from the top surface. The dimensions of the tensile testing specimens are shown in Figure 7b.

**Figure 8:**
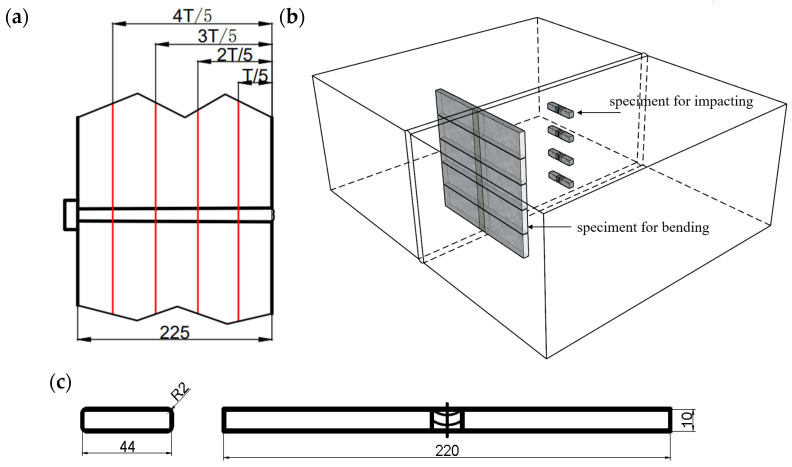
Four different points along the thickness: (**a**) positions of 1/5 T, 2/5 T, 3/5 T and 4/5 T; (**b**) the positions of the bending test and the impacting test specimens; (**c**) the dimensions of the bending specimen (unit: mm).

The experiments were conducted at four different points along the thickness direction of the joint: 1/5T, 2/5T, 3/5T, and 4/5 T, as shown in Figure 8a. Standard impact specimens with dimensions of 10 mm × 10 mm × 55 mm were prepared to evaluate the Charpy V-notched impact toughness at −40 °C. The impact testing was performed according to ISO 9016-2022 [32]. The bending test at room temperature (20 °C) was performed according to ISO 5173-2023 [33]. The layout of the specimens for bending and impacting is shown in Figure 8b. The dimensions of the bending specimens are shown in Figure 8c. The values of tensile strength, bending, and impact toughness were obtained from the average of three repeated tests. 

Metallographic specimens with dimensions of 20 mm × 20 mm × 20 mm were taken at corresponding positions. After grinding and polishing, the microstructure was etched with a 4% nitrate alcohol solution. The microstructures and the fracture morphology of the specimens were observed by optical microscopy (OM, ZEISS Observer A1m, Carl Zeiss, Jena, Germany) and scanning electron microscopy (SEM, HITACHI S-3700N, Hitachi, Tokyo, Japan). The hardness distribution along the cross-section of the weld joint from the low BM to the top BM was measured at 1/5 T, 2/5 T, 3/5 T, and 4/5 T. The hardness distribution of the weld center at corresponding positions was compared. The hardness test was measured by a Vickers micro-hardness machine with a test load of 500 g and a dwell period of 15 s. 

## 3. Results and Discussion

### 3.1. Tensile and Micro-Hardness Testing

**Figure 9:**
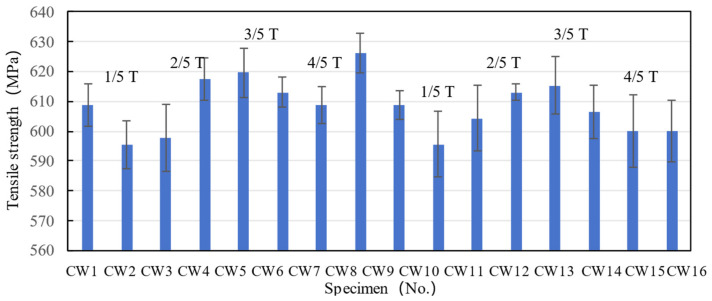
Through-thickness tensile properties of the welding joint.

Figure 9 presents the tensile properties along the thickness using cross-weld tensile specimens. All specimens fractured at the HAZ, indicating that the welding joint has high strength-matching characteristics. The results showed that the tensile strength of all parts of the weld joint was above 595 MPa. The strength decreased at 1/5 T, then increased at 2/5 T and 3/5 T, and then dropped at 4/5 T. The tensile strength at 3/5 T was about 615 MPa, greater than other positions.

**Figure 10:**
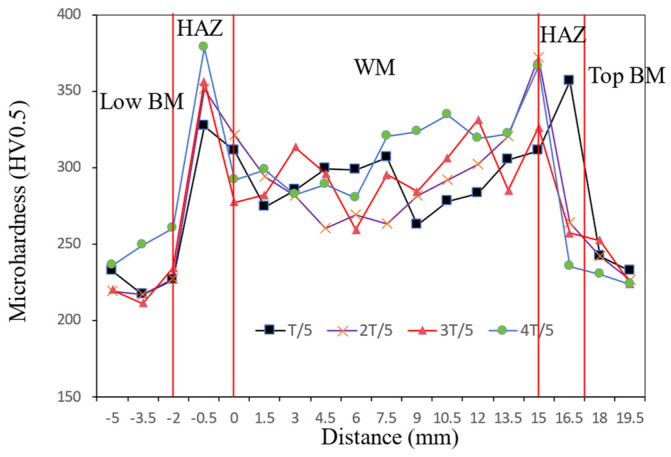
The hardness distribution along the cross-section of the weld joint from low sidewall to top sidewall.

Figure 10 shows the hardness distribution along the cross-section of the weld joint. The HAZ had a higher hardness value than the WM, and the BM presented the lowest hardness. Notably, in the HAZ, there existed significantly higher hardness values, with an average reading of 318.8 HV0.5, indicating hardening phenomena occurring in this region. And the results showed that the hardness of the HAZ was increased by 38% compared with the BM. In addition, the average hardness of the WM was 293.6 HV0.5, which was 27.1% higher than that of the BM. The hardness of the upper WM was higher than the lower WM because of the lower heat input.

**Figure 11:**
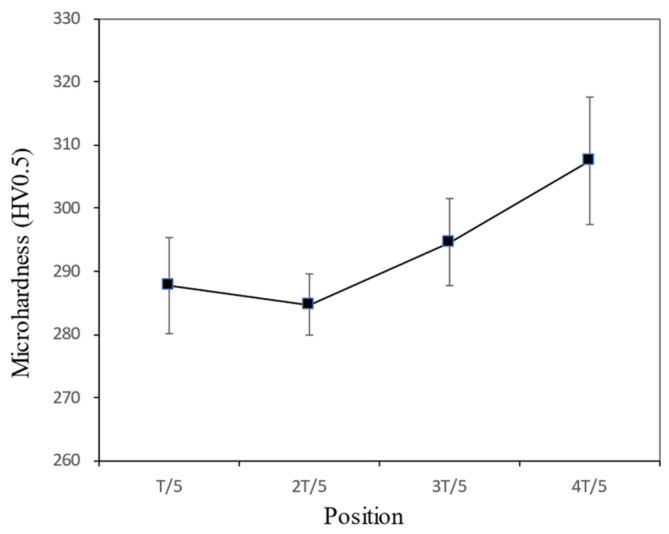
The hardness distribution along the cross-section of the WM.

Figure 11 shows the micro-hardness of the WM at 1/5 T, 2/5 T, 3/5 T, and 4/5 T. Overall, the micro-hardness had the highest values at 4/5 T, compared with those at 1/5 T, 2/5 T, and 3/5 T. In addition, with the increase in thickness from 1/5 T to 2/5 T, the micro-hardness slightly decreased, while from 2/5 T to 4/5 T, the micro-hardness significantly increased from 285 HV0.5 to 307 HV0.5.

### 3.2. Bend and Impact Testing

**Figure 12:**
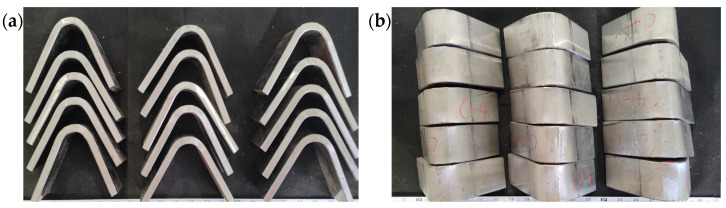
Bending test specimens of weld joint: (**a**) bending angle; (**b**) bending surface.

At room temperature (20 °C), a bending test was performed on S500Q steel weld joints with a bending center diameter of 80 mm and a bending angle of 180°. As shown in Figure 12a,b, there were no microcracks observed in both the weld zone and the HAZ, indicating an excellent plastic deformation capacity and fusion quality throughout the entire weld joint.

**Figure 13:**
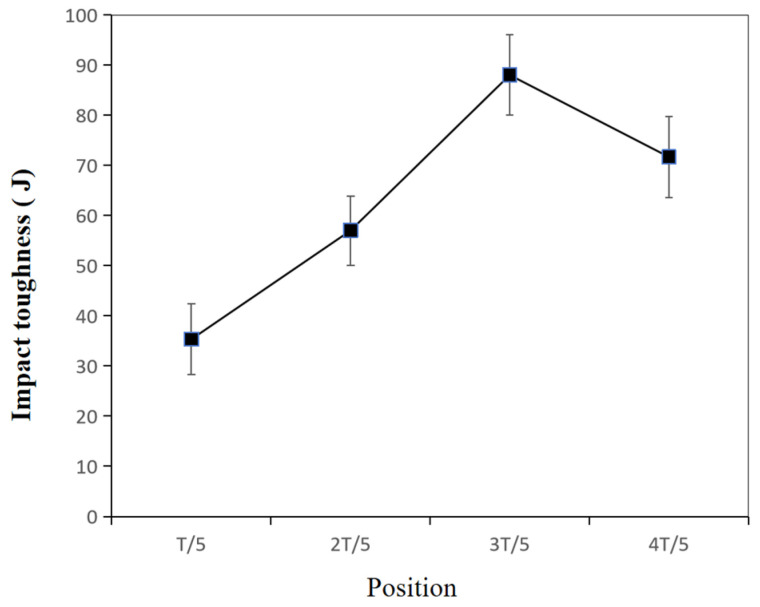
Low-temperature toughness test results of S500Q steel.

Figure 13 shows the impact toughness of the WM at different positions. Overall, the impact-absorbed energy had the highest values at 3/5 T, compared with those at 1/5, 2/5 and 4/5 T. In addition, with the decreasing thickness, the impact-absorbed energy slightly increased from 4/5 T to 3/5 T and then decreased from 3/5 T to 1/5 T. Also, at a testing temperature of −40 °C, the impact-absorbed energy of the WM dropped from 88 J at 3/5 T to 35.3 J at 1/5 T, which was a 59.9% decrease, indicating that the impact toughness was sensitive to the position of the thickness.

### 3.3. Microstructure and Fractography Analysis

**Figure 14:**
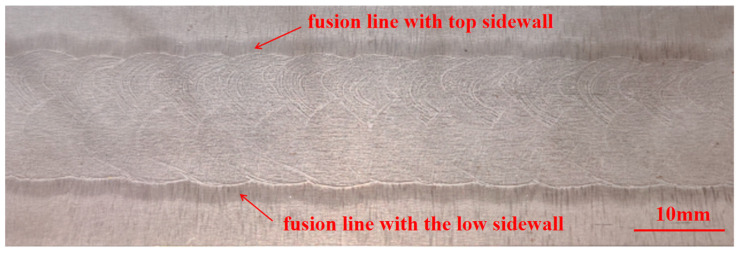
Cross-section macrograph of weld joint.

The macroscopic morphology of the narrow-gap GMAW weld joint of S500Q steel is illustrated in Figure 14. The fusion line had a good fusion quality with the top and the low sidewalls. Each layer of the lower path had a width of about 9.4 mm and a thickness of about 6.8 mm, while the upper path had a width of about 7.4 mm and a thickness of about 6.2 mm. The size of the lower path was larger than that of the upper path. The darker-colored area represents the HAZ, resulting from thermal cycling during the welding process, with its width about 1.7 mm. It can also be seen from Figure 14 that the fusion line with the low sidewall was more optically distinct. This could be attributed to the wider hardened area, due to a higher heat input during the welding process.

**Figure 15:**
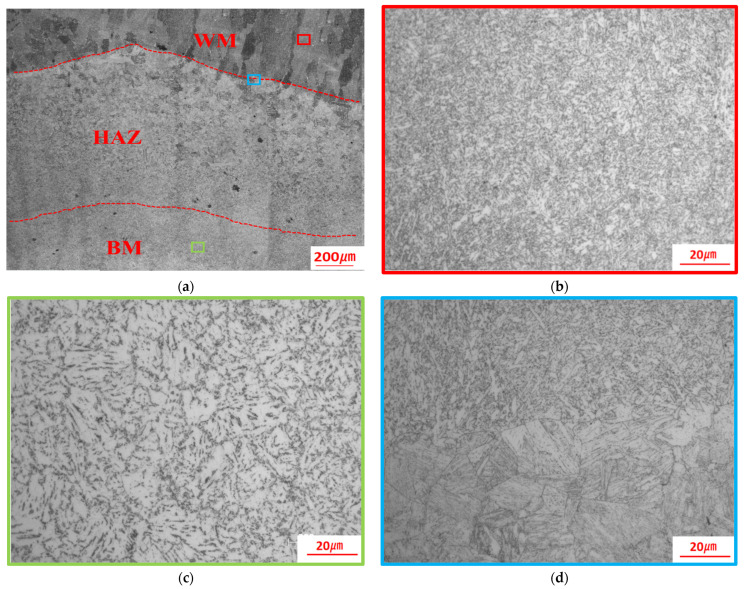
Cross-section macrograph of weld joint: (**a**) microstructure of HAZ; (**b**) microstructure of WM; (**c**) microstructure of BM; (**d**) microstructure of fusion line.

Figure 15 shows the OM microstructure analysis of the weld joint. Figure 15a shows the BM, fine-grained zone, coarse-grained zone, fusion line, and WM in sequence. Figure 15b shows that the microstructure of the WM was composed of granular bainite, tempered soxhlet, and a small amount of upper bainite. Figure 15c shows that the microstructure of the BM was primarily composed of granular bainite, along with small amounts of massive ferrite and sorbite. The inter-crystalline δ-ferrite near the fusion line was heated and coarsened, which resulted in the formation of the HAZ, as shown in Figure 15d. The microstructure of the HAZ was composed of a fine-grained zone and coarse-grained zone. The WM uniformly transitioned to the coarse-grained zone, where the grain boundary was fine and the grain was not obviously coarsened. The coarse-grained zone uniformly transitioned to the fine-grained zone, which was a completely recrystallized zone. The grains in the completely recrystallized zone were uniform and fine. A coarse-grained transition zone between the fine-grained zone and the BM was an incompletely recrystallized zone, and the structure was a mixture of granular bainite and soxhlet, with some tretenite precipitates at the grain boundary. The precipitation was due to the different cooling rates. The tristenite would precipitate along the original austenite grain boundary with a slow cooling rate during the welding process. Figure 15d shows that martensitic structures were evident near the fusion line. Acicular ferrite was predominantly interspersed with minor reticulation in the WM, and the composition was formed by the combination of semi-reticulated first eutectic ferrites and bainite. Acicular ferrites formed nuclei and grew in crystals, and the grain size was refined, thereby improving the toughness of the WM and HAZ.

**Figure 16:**
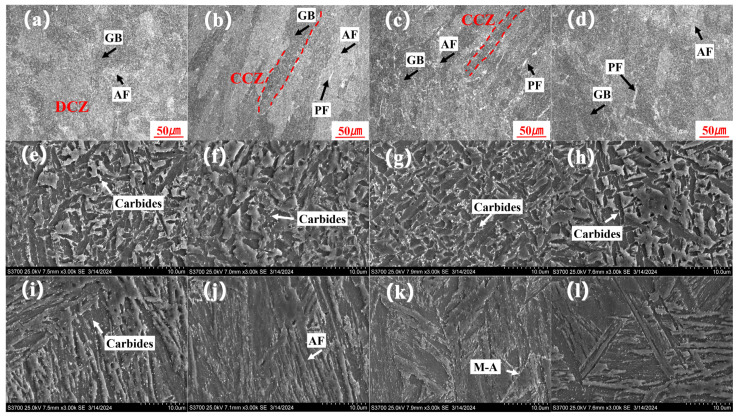
The OM and SEM at various positions of the WM: (**a**–**d**) OM; (**e**–**h**) SEM of WM; (**i**–**l**) SEM of HAZ; (**a**,**e**,**i**) 4/5 T, (**b**,**f**,**j**) 3/5 T, (**c**,**g**,**k**) 2/5 T, (**d**,**h**,**l**) 1/5 T.

Figure 16 presents the OM and SEM images of the weld joint specimens at various positions. As presented in Figure 16a,e,i, the microstructure of the WM at 4/5 T consisted of a few dendritic crystal zones (DCZs), characterized by granular bainite and acicular ferrite. The larger carbides were precipitated in the WM and HAZ along the grain boundaries. In Figure 16b,f,j, the microstructure of the WM at 3/5 T consisted of a few columnar crystal zones (CCZs), characterized by granular bainite, acicular ferrite, and a little pro-eutectoid ferrite, which could be explained by the thermal gradient and the cooling rate. A large number of fine and dispersed granular carbides were precipitated in the grained region. In Figure 16c,g,k, the microstructure of the WM at 2/5 T consisted of a few CCZs, characterized by granular bainite, acicular ferrite, and more pro-eutectoid ferrite. The larger carbides were precipitated in the WM. Martensite–austenite constituents were precipitated at the ferritic grain boundary in the HAZ. In Figure 16d,h,l, the microstructure of the WM at 1/5 T consisted of a few DCZs, characterized by granular bainite, acicular ferrite, and a little pro-eutectoid ferrite. At 4/5 T and 1/5 T, the heat dissipation after welding existed as body heat dissipation and surface heat dissipation. At 3/5 T and 2/5 T, it was mainly based on body heat dissipation, and the direction of the thermal gradient was more obvious, where CCZs were represented. 

In summary, during the welding process of NG-GMAW with low heat input, the cooling rate at the 4/5 T position was correspondingly fast, so a granular bainite structure was formed [34,35]. The hardness at 4/5 T was greater, with a large number of carbides, which was consistent with the micro-hardness results shown in Figure 11. With the increase in the thickness at 3/5 T, the cooling rate decreased. The carbide was dissolved during heating, and then dispersed and precipitated during the cooling process. The fine granular carbides pierced and hindered the movement of the dislocations. Fine granular carbides promoted the nucleation and growth of acicular ferrite, thereby improving the toughness of the joint [36,37]. It was consistent with the impact toughness results shown in Figure 13. There were a large number of dispersed carbide phases at 2/5 T and 3/5 T playing a dispersion-strengthening role, and the tensile strength of the weld increased. It was consistent with the tensile strength results shown in Figure 9. When the thickness of the joint continued to increase, close to the welding surface, the cooling rate increased, and the carbides were reduced. Coarse granular bainite reduced the toughness at 1/5 T.

**Figure 17:**
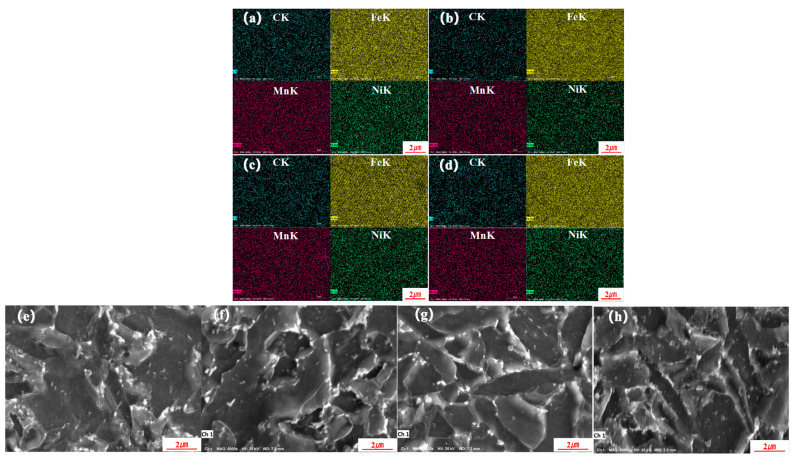
EDS-mapping analyses: (**a**,**e**) 4/5 T, (**b**,**f**) 3/5 T, (**c**,**g**) 2/5 T, (**d**,**h**) 1/5 T.

A energy-dispersive spectrometer (EDS) was further utilized to analyze the microstructure at various positions. Figure 17 shows the EDS mapping of the materials at various positions. From Figure 17a–d, it is clear that the elemental distribution, Fe, Mn, Ni, was uniform. The four positions exhibited numerous carbides, but the density of the carbides at 3/5 T was substantially lower than that at 1/5 T, 2/5 T, and 4/5 T, as shown in Figure 17b,f. And the carbides at 3/5 T were more diffuse. The characteristics were more obvious along the grain boundaries at 1/5 T, 2/5 T, and 4/5 T. The type of carbide was mainly Fe3C [28,38].

**Figure 18:**
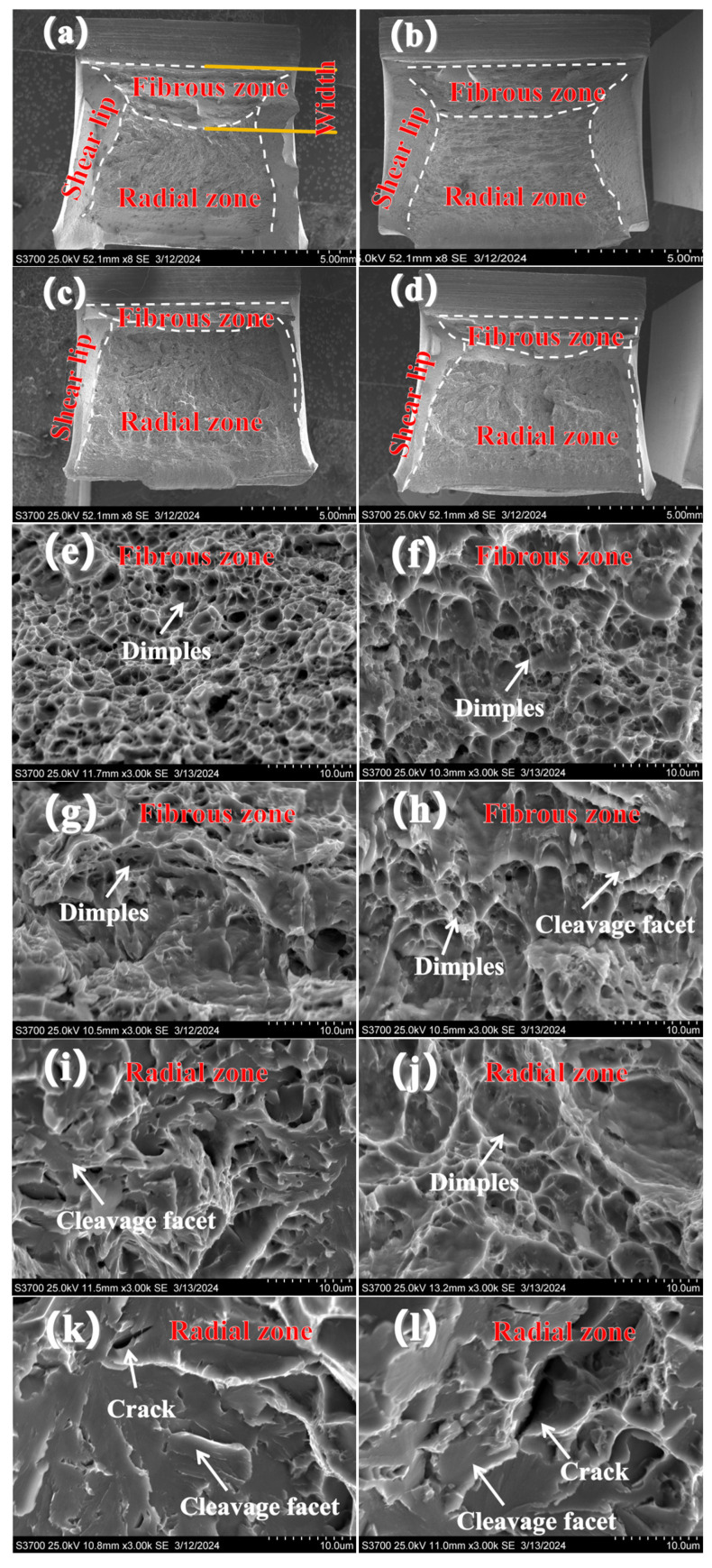
SEM micrographs of the −40 °C impact fracture: (**a**,**e**,**i**) 3/5 T WM; (**b**,**f**,**j**) 3/5 T HAZ; (**c**,**g**,**k**) 1/5 T HAZ; (**d**,**h**,**l**) 1/5 T WM.

A microscopic fracture of 1/5 T and 3/5 T was further analyzed. Figure 18 displays the impact fracture morphologies of the WM and HAZ at 1/5 T and 3/5 T. The fracture surface exhibited fibrous zones, radial zones, and shear lips, as shown in Figure 18 a–d. In the WM, as shown in Figure 18a,e,i, the fibrous regions at 3/5 T contained more deep and big dimples. The fibrous zones at 1/5 T displayed shallow and small dimples, along with cleavage facets on the fibrous zone, as shown in Figure 18d,h,l. In the HAZ, as shown in Figure 18b,f,j, the fibrous regions at 3/5 T contained more deep and big dimples. The fibrous zones at 1/5 T displayed shallow and small dimples, along with cleavage facets on the fibrous zones, as shown in Figure 18c,g,k. The presence of large and deep dimples indicated that more energy would be consumed for deformation. The fibrous zones at 3/5 T accounted for 21.9% of the WM and 26.4% of the HAZ. The fibrous zones at 1/5 T accounted for 14.8% of the WM and 10.6% of the HAZ. It was observed that the width of the fibrous zone at 3/5 T was significantly larger than that at 1/5 T. Wider fiber zones are less prone to destabilize fractures in the material. This means that the higher the proportion of the fibrous zone, the better the impact toughness of the material. It was considered that the change in fibrous zone areas and fracture mode could be attributed to the presence of different microstructures and precipitations. A higher impact toughness was observed in the presence of a fine carbide distribution and acicular ferrite microstructure.

In this study, the radial zones at 1/5T were observed to exhibit cleavage, with secondary cracks on the fracture surface, indicative of a brittle fracture mode. However, the radial zone at 3/5 T was found to display a ductile fracture with dimples and an absence of secondary cracks on the fracture surface. 

## 4. Conclusions

In this study, the microstructure and mechanical properties of a high-depth-to-width-ratio horizontal NG-GMAW joint, through the entire thickness of S500Q steel, were studied. The main conclusions are as follows:

Compared with the 1/5 T, 2/5 T and 4/5 T positions, the 3/5 T position had the highest strength, with a tensile strength reaching about 615 MPa. This was attributed to the formation of finer carbide precipitates.

At −40 °C, the impact toughness of the WM decreased by 59.9% from 88 J at 3/5 T to 35.3 J at 1/5 T, indicating that the impact toughness is sensitive to the thickness position. The microstructure at 3/5 T was mainly granular bainite, acicular ferrite, and fine dispersed granular carbides. The microstructure at 1/5 T was mainly coarse granular bainite, acicular ferrite, and a little pro-eutectoid ferrite. The coarse granular bainite was the essential reason for the decrease in impact energy.

The fibrous zone of the impact fracture at 3/5 T showed a ductile fracture. The radiation zone at 1/5 T showed a combination of a brittle fracture and a ductile fracture. 

## Figures and Tables

**Table 1:** Mechanical properties of S500Q steel.

Yield Strength (MPa)	Tensile Strength (MPa)	Elongation (%)	Impact Energy at −40 °C (J)
470~492	576~590	19.5~24	77~193

**Table 2:** Chemical compositions of S500Q steel.

C	Si	Mn	P	S	Ni	Cr	Ti	Mo	Als	Nb	Fe
0.09 ~0.16	0.15 ~0.45	0.08 ~1.45	≤ 0.012	≤ 0.003	0.8 ~1.3	0.35 ~0.65	0.010 ~0.030	0.35 ~0.65	0.015 ~0.050	0.015 ~0.045	balance

**Table 3:** Chemical composition of AWS ER90S-G welding wire.

C	Si	Mn	S	P	Ni	Mo	Cu	Fe
0.09 ±0.016	0.54 ±0.108	1.51 ±0.025	0.008 ±0.0023	0.008 ±0.0006	0.98 ±0.156	0.25 ±0.117	0.12 ±0.029	balance

**Table 4:** Mechanical properties of AWS ER90S-G welding wire.

Yield Strength (MPa)	Tensile Strength (MPa)	Elongation (%)	Impact Energy at −40 °C (J)
601 ± 34	685 ± 35	26 ± 3	104 ± 11

**Table 5:** Welding parameters.

Lower Path Welding Current (A)	Upper Path Welding Current (A)	Welding Voltage (V)	Welding Speed (cm/min)	Gas Flow Rate (L/min)	Wire Extension (mm)	Inter-Pass Temperature (°C)	Contents of CO_2_ in Shielding Gas
260	240	26	20	26	15–17	130–150	22%

## Data Availability

Data are contained within the article.

## Nomenclature

NG-GMAW	narrow-gap gas metal arc welding
GMAW	gas metal arc welding
SEM	scanning electron microscopy
WM	weld metal
HAZ	heat-affected zone
BM	base metal
OM	optical microscopy
DCZs	dendritic crystal zones
CCZs^du^	columnar crystal zones
EDS	energy-dispersive spectrometer
CW	cross-weld tensile specimen
1/5T	1/5 thickness from weld surface
2/5T	2/5 thickness from weld surface
3/5T	3/5 thickness from weld surface
4/5 T	4/5 thickness from weld surface
M-A	martensite-austenite constituents
